# Influence of Flame Retardant Impregnation on Acoustic and Thermophysical Properties of Recycled Technical Textiles with the Potential for Use in Wooden Buildings

**DOI:** 10.3390/polym13162598

**Published:** 2021-08-05

**Authors:** Anna Danihelová, Patrik Sčensný, Tomáš Gergeľ, Vojtěch Ondrejka, Miroslav Němec, Rastislav Igaz, Jozef Štefko, Iveta Mitterová

**Affiliations:** 1 Department of Fire Protection, Faculty of Wood Sciences and Technology, Technical University in Zvolen, T.G. Masaryka 24, 960 01 Zvolen, Slovakia; danihelova@acoustics.sk (A.D.); scensnypatrik@gmail.com (P.S.); mitterova@tuzvo.sk (I.M.); 2 National Forest Centre, Forest Research Institute, T.G. Masaryka 22, 960 01 Zvolen, Slovakia; tomas.gergel@nlcsk.org (T.G.); vojtech.ondrejka@nlcsk.org (V.O.); 3 Department of Physics, Electrical Engineering and Applied Mechanics, Faculty of Wood Sciences and Technology, Technical University in Zvolen, T.G. Masaryka 24, 960 01 Zvolen, Slovakia; igaz@tuzvo.sk; 4 Department of Wooden Constructions, Faculty of Wood Sciences and Technology, Technical University in Zvolen, T.G. Masaryka 24, 960 01 Zvolen, Slovakia; stefko@tuzvo.sk

**Keywords:** technical textiles, flame retardant, sound absorption, thermal conductivity, thermophysical properties

## Abstract

This article presents the results of an investigation of acoustic and thermophysical properties of insulation panels made from recycled technical textiles originating from the automotive industry. Measurements were performed on the samples of insulation panels (Senizol AT XX2 TL60), which were modified with liquid flame retardants (ISONEM^®^ ANTI-FIRE SOLUTION, ECOGARD^®^ B45, HR Prof). Another method of treatment was carried out by surface application of non-flammable facing (woven carbon fibre, nonwoven carbon fibre). Retardants were applied to the samples by surface spraying and soaking. The results showed a high ability of material to absorb sound in the frequency range 350 Hz–2 kHz. The sound absorption coefficient ranged from 0.82 to 0.9 in the frequency range 500 Hz–2 kHz. The noise reduction coefficient is 0.75. After material modification with the flame retardants, there was no significant change of sound absorption. The thermal conductivity coefficient of material before modification was 0.038 W⋅m^−1^⋅K^−1^. After application of the flame retardants, the thermal conductivity coefficient increased depending on type and method of retardant application in the range of 2.6–105.3%. The smallest change was detected after modification of material with ECOGARD^®^ B45.

## 1. Introduction

In recent decades, society has been facing constant environmental problems and challenges, such as global warming, ozone depletion and waste accumulation. Effective measures must be sought and implemented to prevent these phenomena from getting worse. The construction industry plays an important role in energy consumption, waste production and generation of air emissions. Buildings have a very significant part in energy consumption in particular phases of their life cycle. Current trends in sustainability are leading towards the transformation of the material sector towards ecologically friendly and natural materials including wood, as well as composites based on eco-friendly materials. Overall consumption must include all energy from production and distribution of materials, construction and use to the eventual disposal of the building and generated waste [1].

One of the most important challenges in the construction of new buildings is the reduction of energy consumption in all mentioned phases. According to a study by the United Nations Environment Programme, it is estimated that 40% of the world’s energy resources, 25% of water resources and 40% of global material resources are used in the operation of buildings. In addition, the buildings life cycle is responsible for a third of the world’s greenhouse gas production [2,3].

Reducing energy consumption of buildings is achieved by insulating them with thermal insulation materials. With a change of investors´ demands and as a result of new legislative requirements, the emphasis is placed not only on thermophysical properties of material, but also acoustic and fire properties are important for building materials. By choosing a suitable insulation material and right applications, it is possible to reduce energy consumption by up to 50–70% [4].

One of the ways to reduce the negative footprint is the construction of wood-based buildings. Due to economic requirements, the proportion of wood that provides a static function in these construction systems is relatively low. On the contrary, space was given up for filling and cladding materials, which provide mainly thermal insulation, sound insulation and fire protection function. The typical composition of the panel or timber-frame construction of the external wall or roof of the current low-energy and almost-zero-energy houses amounts to approximately 30 cm of thermal insulation filling, which ultimately leads to considerable volumes of insulation in the building itself.

Commonly used materials for thermal insulation are mineral fibres or polystyrene and polyurethane boards [5]. Other materials belong to the group of so-called non-traditional insulating materials. These are divided into environmentally friendly and recycled materials. Nature-friendly materials include sugar cane, corn cob, wool, palm fibre, rice, straw, hemp, cork, etc. Nowadays, thermal insulations made from natural materials is used often in structural elements of timber buildings. They act not only as thermal insulation but also as soundproofing; however, they can also provide a required fire-resistance function on the condition that the construction (e.g., external wall) has a suitable assembly [6]. Recycled materials include glass wool, plastics, textiles, wood waste, agricultural crops and more [2,7,8,9,10].

Insulation materials based on recycles materials due to comparable thermal and acoustic properties can fully replace conventional mineral fibre materials and foam materials. Insulation materials made from waste help to solve problems of increasing energy consumption for heating and waste accumulation. Such materials also include treated recycled technical textiles. This waste is currently mostly accumulated in landfills or incinerated in special waste incinerators. Use of these materials after their lifespan is one of the European Union´s requirements for waste management (EU´s Approach to Waste Management). According to Eurostat statistics, in 2017 the utilisation rate of recycled materials accounted for 11.2% of raw material resources in the EU, but in Slovakia it was only 4.9% [11].

It is difficult to decompose materials used in the automotive industry because they consist of hardened fabrics and combined textile and non-textile components. These materials are not suitable for further textile processing [12]. Wang declared that the system of technical textile recycling is not sufficient and one of the reasons is a small possibility of reuse [13].

Nowadays, recycling of textiles as well as other materials is a highly discussed topic. A significant part of the research is devoted to the recycling of materials and their subsequent use in the production of insulation materials for construction [14,15,16]. Research of insulating panels is focused mainly on their thermophysical, acoustic, hydrothermal, fire and environmental properties [17,18,19].

The recycling process of technical textiles used for production of insulation panels consists of several steps. First, waste is sorted by hand to clean it of unwanted materials, then it is crushed and defibered. Material treated this way still need to be loosened, homogenised or further steps can be done according to the requirements of the end use [20].

**Figure 1:**
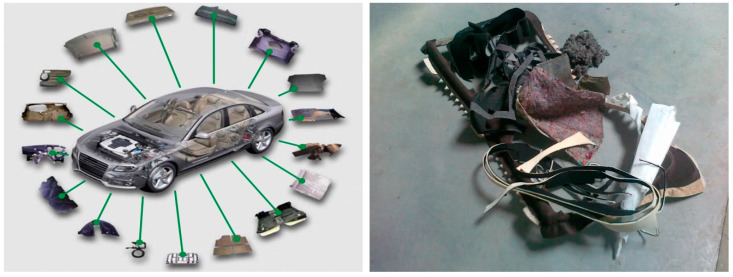
Recycled automobile parts [12].

Our research is focused on textile material used in the automotive industry (technical textiles). Mass of these materials used in the car (Figure 1) represents approximately 3% of total automobiles mass [21]. The waste of technical textiles from the cars appears to be interesting for construction [22]. Great attention is already paid to the properties of technical textiles before their use in automobiles. It is primarily about their ability to reduce noise and vibration. They also increase thermal comfort. Similar requirements apply to the materials used in construction. This means that material made from the recycled technical textiles will have a potential to be an effective isolating material.

Flame retardants are applied to these materials to improve their fire properties. These are substances which, in a chemical, physical or combined way, prevent rapid ignition and combustion [23,24]. Due to the high flammability of textile materials used for upholstery and other applications, standards are set to minimise possible losses caused by fire of these materials. To achieve the required fire safety, products are modified with chemicals that prevent not only ignition but also flame spread at various stages of combustion process.

The aim of this work was to assess the influence of selected flame retardants on acoustic (sound absorption coefficient) and thermophysical properties (thermal conductivity, thermal diffusivity coefficient and specific heat capacity) of insulating materials made of the recycled technical textiles. 

## 2. Materials and Methods

Research of thermophysical and acoustic properties was carried out on a material called Senizol AT XX2 TL60 (Stered PR Krajné s.r.o., Krajné, Slovakia), which consists of a synthetic and natural fibre mixture originating from the automotive technical textiles (car seats, ceiling upholstery, anti-noise upholstery, foot pads, etc.) from cars interconnected by polyurethane adhesive and water. Polyester, polypropylene and polyurethane have the largest share in material composition, followed by polyaramid and polyethylene [20]. Insulation panels with a thickness of 60 mm were used for modification of samples.

The material was tested in its original form (without flame retardants) and subsequently after application of three types of flame retardants. Retardants were applied in two ways. These two ways of application of the flame retardants are standard and they are also recommended by the manufacturers. Due to this reason, another aim of the research was to find out how the method of retardant application affects the acoustic and thermophysical properties. In the first case, the flame retardants were applied to samples by spraying (coating mass was HR Prof 60 g m^−2^, ECOGARD^®^ B45 was 66 g m^−2^ and ISONEM^®^ ANTI-FIRE SOLUTION was 77 g m^−2^). In the second case, the samples were immersed into the retardation solution for 5 s. Flame retardant consumption of ISONEM^®^ ANTI-FIRE SOLUTION was 430 g m^−2^, HR Prof was 532 g m^−2^ and ECOGARD^®^ B45 was 542 g m^−2^. Subsequently, the samples were conditioned at air temperature 20 °C and relative humidity 50% for 30 days.

Commercially available retardants ISONEM^®^ ANTI-FIRE SOLUTION, ECOGARD^®^ B45, HR Prof were used to modify material. Their composition is the know-how of manufacturers, which cannot be published.

The solution for fire protection ECOGARD^®^ B45 (TÜCHLER Bühnen-& Textiltechnik GmbH, Wien, Austria) is a universally applicable impregnating agent designed for all absorbent natural materials and many synthetic materials that protect against common ignition sources. It is characterised as a colourless, odourless aqueous solution that contains phosphorus and halides. ECOGARD^®^ B45 is not self-ignitable and is not explosive. This flame retardant provides physical protection against flames and prevents the formation of salt [25].

ISONEM^®^ ANTI-FIRE SOLUTION (ISONEM BOYA VE YALITIM TEKNOLOJILERI INS. SAN. TIC. A.S., Izmir, Turkey) is characterised by the manufacturer as a substance that provides absolute resistance to fire. This transparent soaking solution can be applied to wood, textiles, wool, cotton, polystyrene and other combustible materials. ISONEM^®^ ANTI-FIRE SOLUTION, the non-flammability solution, encapsulates the applied surface as molecules and prevents its contact with oxygen. Thanks to its active ingredients, it prevents it from reaching the temperature that will initiate the combustion reaction. In this way, the substance to which it is applied never catches fire. ISONEM^®^ ANTI-FIRE non-flammability solution is a product that is produced from 100% natural materials, has no harm to human health and is anti-bacterial [26].

HR Prof (Holz Prof, Tallin, Estonia) is designed for modification of wooden structures, stairs, coffered ceilings, wooden floors and other wood and cellulose products in order to ensure their resistance to ignition under direct flame. It is a light brown, odourless liquid. Active substance in this agent is ferric phosphate (30%). HR Prof, which possesses high diffusion properties, quickly penetrates the structure of the substrate. Once absorbed into the surface of the material, HR Prof combines chemically within the cell structure but does not form a surface finish. Materials treated with HR Prof when exposed to temperatures of up to 1700 °C are subject to charcoal forming, severely restricting the spread of flame [27].

Acoustic properties of materials were also studied with surface application of carbon woven and nonwoven fibres. Insertion of carbon fibres into the composition of material can increase tensile strength of the insulation panel, but it also reduces its mass and increases thermal resistance [28]. The disadvantage is its relatively high price (Kordcarbon, a.s., Strážnice, Czech Republic).

### 2.1. Acoustic Properties

**Figure 2:**
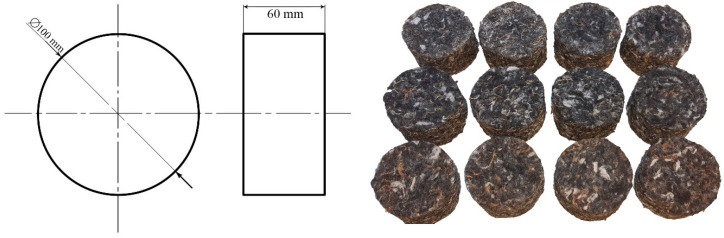
Dimensions of samples for sound absorption coefficient measurement.

Measurement of the sound absorption coefficient was performed by using circular cross section samples with a diameter of 100 mm and a thickness of 60 mm (Figure 2). Four test specimens were made for each modification.

Measurement of sound coefficient was performed according to ISO 10534-2 [29]. The test method covers the use of an impedance tube, two microphones and a digital frequency analysis system. This two-microphone method of measuring sound absorption is based on decomposition of a broadband random signal into a signal from a source and a reflected signal. The complex acoustic transfer function is calculated from obtained values of acoustic pressure. From these values is possible to determine sound absorption coefficient and reflectance coefficient for all frequencies [30].

**Figure 3:**
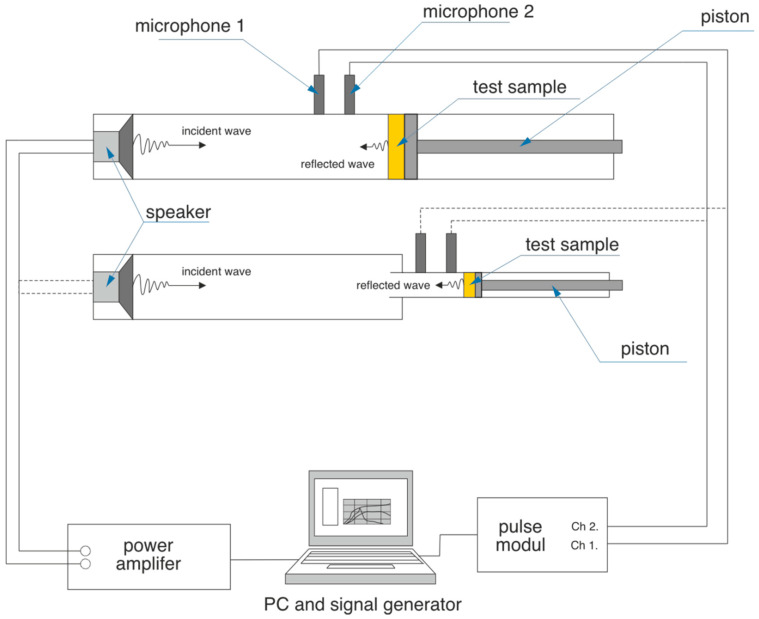
Scheme of device for measuring of sound absorption coefficient.

The measuring device consists of an impedance (Kundt´s) tube Brüel & Kjær type 4206, a PULSE 14 system, on LAN-XI module Brüel & Kjær type 3050 with two active inputs and CPB (Constant Percentage Band), a sound signals generator, two identical microphones and a computer to display and save measured data (Figure 3) (Brüel & Kjær an HBK company, Nærum, Denmark). Measurement of sound absorption coefficient was performed in frequency range of 20 Hz to 2 kHz, using Kundt´s tube with a diameter of 100 mm.

Another investigated parameter was the Noise Reduction Coefficient (NRC). It is a single-number rating which represents the average of sound absorption coefficients of a material at specific mid-range frequencies (tested at 250, 500, 1000 and 2000 Hz octaves) rounded to nearest 0.05. This value is influenced by thickness and density of material [31].

### 2.2. Thermophysical Properties

**Figure 4:**
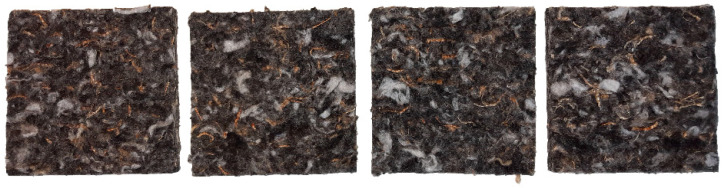
Samples for measurement of thermophysical properties by EDPS method.

Thermophysical properties were measured on samples with dimensions 100 mm × 100 mm and thickness of 13 mm (Figure 4). From total number of 42 samples, 21 pairs were formed for determination of thermophysical properties. Three pairs of samples were determined for each modification, with five repeated measurements taken on each pair. The set of measurement results for each modification method includes 15 determined values.

**Figure 5:**
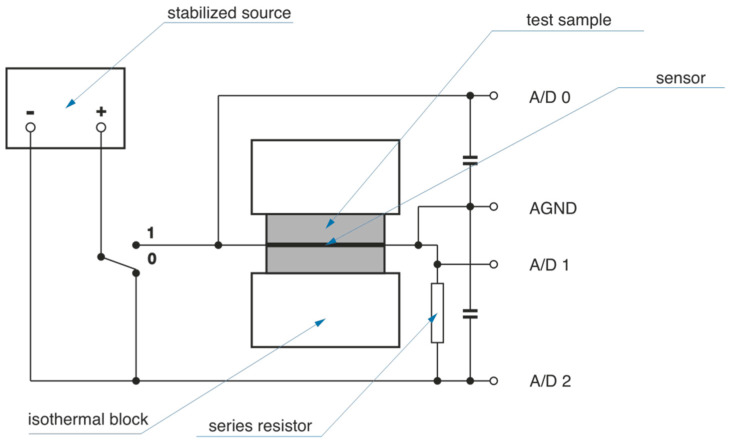
Scheme of EDPS method apparatus [32].

The EDPS (Extended Dynamic Plane Source) method was used to measure thermophysical properties. It is one of the non-stationary methods for this purpose [32,33]. The EDPS method is based on the Dynamic Plane Source (DPS) method, andit can be used to investigate the thermal conductivity, thermal diffusivity and specific heat capacity of low-conductivity materials (λ ≤ 2 W·m^−1^·K^−1^) [34,35]. Figure 5 shows the device scheme.

The measurement process is performed by supplying heat between a set of two small-thickness samples, thereby simulating a one-dimensional heat diffusion process. Heat is supplied through a resistance film, which also measures temperature. Thermal conductivity and thermal diffusivity of the samples are determined on the basis of the time change of temperature of the heating foil in time using the Equation (1):

(1) $$T\left(t\right)=\frac{qh}{\lambda }\cdot \sqrt{\frac{t}{\pi \Theta }}\left(1+2\sqrt{\pi }\overset{\infty }{\underset{n=1}{\sum }}\beta^{n}\mathrm{ierfc}\left(n\sqrt{\frac{\Theta }{t}}\right)\right)$$

where *q* is the heat flux (W⋅m^−2^),

*λ* is the thermal conductivity (W⋅m^−1^⋅K^−1^),

Θ is the characteristic time defined by Equation (2),

(2) $$\Theta =\frac{d^{2}}{a}$$

*d* is material thickness (m),

*a* is thermal diffusivity coefficient of material (m^2^∙s^−1^).

The specific heat capacity *c* (J·kg^–1^·K^–1^) of the material is determined on the basis of known density *ρ* (kg·m^–3^) and thermal diffusivity coefficient according to Equation (3):

(3) $$c=\frac{\lambda }{a\cdot \rho }$$

## 3. Results and Discussion

### 3.1. Results and Discussion of Acoustic Properties

Different requirements are placed on building structures. Besides thermophysical properties there are also sound insulation properties, which depend on structure and composition of used insulation material. On the other hand, it is important to create acoustic comfort in any enclosed space. Acoustic comfort can be achieved by modifying the interior with suitable materials, which can absorb part of acoustic energy affecting the interior acoustic. Our previous research [22,36,37] focused on sound absorption evaluation of Senizol AT XX2 TL60 have shown its high sound absorption capacity. Sound absorption coefficient α in frequency range 500 Hz–2 kHz reached values from 0.82 to 0.93 and sound reduction coefficient (NRC) was 0.75. The aim of research was to determine effect of treatment with chosen flame retardants on sound absorption of Senizol AT XX2 TL60. Based on the results of our measurements, material Senizol AT XX2 TL60 before treatment as well as after each treatment can be characterised as “very highly absorbent” according to ISO 11654:1997 and classified in sound absorption class A [38].

**Figure 6:**
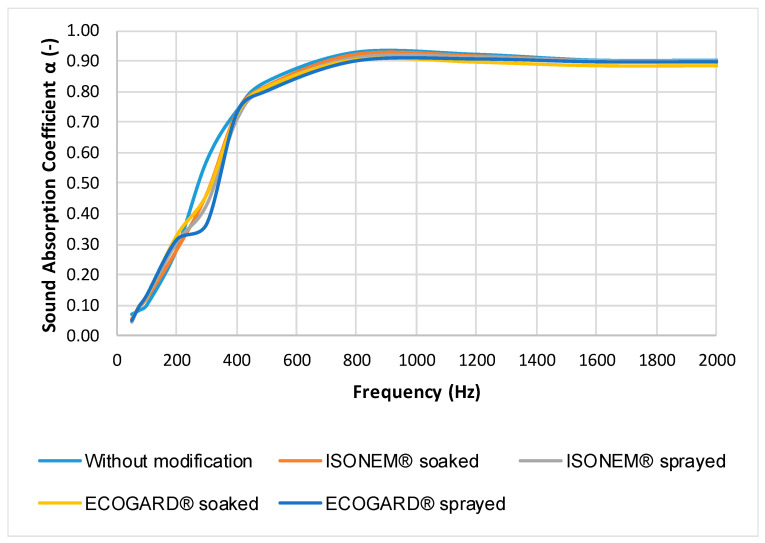
Frequency dependence of sound absorption coefficient α (-) of Senizol AT XX2 TL60 before and after application of ECOGARD^®^ B45 and ISONEM^®^ ANTI-FIRE SOLUTION.

**Figure 7:**
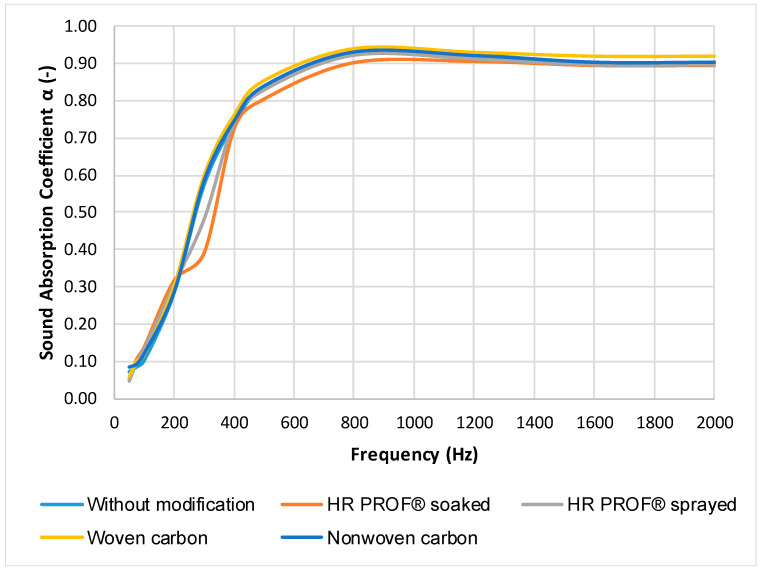
Frequency dependence of sound absorption coefficient α (-) of Senizol AT XX2 TL60 before and after application of HR Prof, woven and non-woven carbon foil.

Frequency dependence of sound absorption coefficient of Senizol AT XX2 TL60 after treatment with flame retardants is presented in Figure 6 and Figure 7. Measurement was performed in the laboratory entitled “Centre of Acoustic Telematics Systems Technical University in Zvolen” at 20 °C temperature and 50% relative humidity. Measurement was performed in frequency range 20 Hz–2 kHz. This frequency range is sufficient because the sound absorption coefficient at the frequencies above 2 kHz was constant (approximately 0.9).

From the graphs in Figure 6 and Figure 7, it is evident that no modification of Senizol AT XX2 TL60 with selected flame retardants caused a significant worsening sound absorption of the investigated material. It can be stated that the dependence curves of sound absorption coefficient on frequency of material made from technical textiles before and after modification with flame retardants are almost identical. The greater effect of ECOGARD^®^ B45 flame retardants and ISONEM^®^ ANTI-FIRE SOLUTION on the sound absorption coefficient was demonstrated only in the frequency range of 200 Hz to 400 Hz. The fire retardants are waterproof but vapor permeable. This property allows the flow of air through the material without significant change, so the treatment with flame retardants will minimally affect the sound absorption of the material. The retardants cover the surface of the fibres of the material, but there are no significant changes in the volume of air between the fibres. It is clear from Figure 7 that change in the sound absorption coefficient was recorded after treatment of the material with the HR Prof flame retardant (after both application methods) in the frequency interval 200 Hz–1 kHz. HR Prof forms a thin oily layer on the fibres, and the surface area of the fibres increases and the porosity decreases. The sound absorption performance decreases with decreasing porosity, which is probably the cause of lower sound absorption after both methods of application of HR Prof retardant. The NRC value was 0.75 for all samples.

Tiuc et al. created a panel made from textile waste (40%) and polyurethane foam (60%) [39]. The NRC of this material was 0.6. Similar research was performed by Buratti et al., who examined sound absorption of insulation panels consisting of a 5 mm recycled paper layer and a 45 mm layer of wool fibres. The NRC value of 0.6 was also found for this material [40].

Tiuc et al. studied the sound absorption coefficient of material made of textile waste mixed with rigid polyurethane foam. The textile waste contained synthetic fibres from the production of knitted clothing, with a density of 0.03 g⋅cm^−3^. The proportion of textile waste in the material was 10%, 15% and 20%. Results showed that sound absorption coefficient improves in frequency interval 100–850 Hz in the case of 80% rigid polyurethane foam and 20% textile waste. The maximum value of sound absorption coefficient was 0.5 at 800 Hz. However, sound absorption decreased at lower and higher frequencies. On the other hand, the sound absorption ability of material with 10% textile waste content increased in the frequency interval 1.1–2.8 kHz in comparison with the 100% rigid polyurethane foam material. In this case, maximum value of sound absorption coefficient was 0.7 at 1.2 kHz [41].

Rubino et al. created composite panels made of 100% wool waste fibres and bound by means of either a chitosan solution or a gum Arabic solution. Acoustic results show absorption coefficients that, for the given thickness, were generally higher than 0.5 from 500 Hz on, and higher than 0.9 from 1 kHz on [18].

Lyu et al. studies studied the effect of flame retardants on the sound absorption coefficient of kapok fibre composites. Under the optimal process parameters, the maximum sound-absorption coefficient reached 0.830, the average sound-absorption coefficient was 0.520, and the sound-absorption band was wide. Therefore, the composites belonged in the category of a high-efficiency sound-absorbing material [42].

If we compare the thermal and acoustic properties of the material made from textile waste Senizol AT XX2 TL60 with commonly used thermal insulation materials (extruded polystyrene, polystyrene, mineral wool), we can see that the thermal conductivity is almost identical, but the sound absorption is different. The thermal insulation performance of EPS (Expanded polystyrene) and XPS (Extruded polystyrene) with similar densities are quite similar, too, but the sound absorption coefficients are different. The sound absorption coefficient of EPS (of comparable thickness 50 mm) has a high sound absorption (α = 0.88) only in a narrow interval around the frequency of 1.3 kHz. XPS has the highest absorption properties in the range of 500 Hz–1 kHz. Mineral wool (thickness 50 mm) has a higher sound absorption coefficient (α = 0.80) at the frequencies from 600 Hz to 1.6 kHz [43]. Measurements showed that the material Senizol AT XX2 TL60 appears to not only be a good thermal insulator, but also has a high ability to absorb sound in a wide frequency range 350 Hz–2 kHz.

### 3.2. Results and Discussion of Thermal Properties

**Figure 8:**
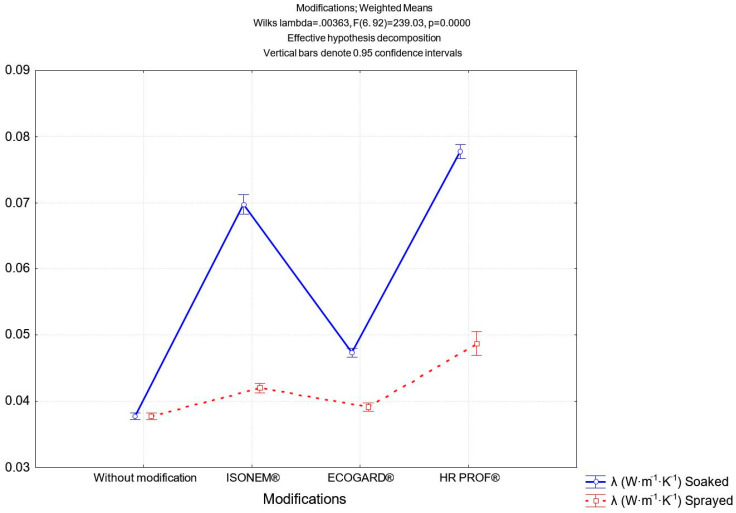
Statistical comparison of thermal conductivity coefficient before and after modification with flame retardants.

The energy consumption for heating in the buildings depends also on the thermophysical properties of the materials that compose the building envelope. Thermal insulation is one of the best ways to reduce the energy consumption due to both winter heating and summer cooling [44]. The primary aim is to reach the lowest possible thermal conductivity. In addition, it is important to know other thermal properties, namely, thermal diffusivity coefficient and specific heat capacity. These properties of the material Senizol AT XX2 TL60 before and after modification with flame retardants were determined in our research. The results of measurements are given in Table 1 (the values and standard deviations of these quantities are gradually found in the individual columns: density *ρ*, thermal conductivity *λ*, thermal diffusivity coefficient *a*, and specific heat capacity *c.* The statistical comparison of thermal diffusivity coefficients before and after modification with selected flame retardants is presented in Figure 8.

The results of Senizol AT XX2 TL60 thermophysical properties measurements show that the thermal conductivity *λ* without modification with flame retardants is 0.038 W⋅m^−1^⋅K^−1^. It can be stated that material made of technical textiles is an adequate alternative to commonly used insulating materials, such as polystyrenes (*λ* = (0.031 W⋅m^−1^⋅K^−1^–0.038 W⋅m^−1^⋅K^−1^)) or fibrous insulating materials based on glass or minerals (*λ* = (0.031 W⋅m^−1^⋅K^−1^–0.045 W⋅m^−1^⋅K^−1^)).

Statistical analysis showed (Figure 8) that due to small variances of measured thermal conductivity coefficients, in all cases of treatment there were statistically significant changes in thermal conductivity of material after modification.

The results of our research showed that the studied material made from technical textiles in its original form has a value of specific heat capacity *c* = 1559.4 J⋅kg^−1^⋅K^−1^, which is in the heat capacity value range of commonly used building insulation materials (800–2060 J⋅kg^−1^⋅K^−1^) (STN 73 0540 -2 + Z1 + Z2) [45]. Senizol AT XX2 TL60, due to its density and heat capacity, can accumulate a lot of heat, which contributes to better thermal stability of buildings interiors. Compared to glass wool–based insulations, which usually have a lower density, this material contributes to the prevention of summer overheating of building interiors, especially wooden buildings.

After Senizol AT XX2 TL60 modification by spraying with flame retardants, density and thermal conductivity increased in all cases. The smallest increase was noticed after application of the ECOGARD^®^ B45 solution (+2.6%). The other two retardants caused a more significant increase in thermal conductivity: 10.5% (ISONEM^®^ ANTI-FIRE SOLUTION) and 29.0% (HR Prof). Other thermophysical properties of samples could not be determined because the EDPS method as a non-stationary method does not allow to determine thermal diffusivity and consequently heat thermal capacity of materials with significant inhomogeneity in thickness of samples. This inhomogeneity was achieved by one-sided spraying of retardant that was diffused into the material.

After soaking the samples in retardants with whole volume, there were significant changes in all monitored thermophysical properties. In addition, density of material changed significantly. In all cases, it increased approximately twofold in comparison to density of original material.

Application of retardation solution by dipping significantly increased thermal conductivity of all samples. The smallest increase was caused by ECOGARD^®^ B45 solution (+23.8%). The other two solutions caused an increase of 84.2% (ISONEM^®^ ANTI-FIRE SOLUTION) and 105.3% (HR Prof) in thermal conductivity. In case of full-volume application of retardants, the thermal conductivity changes to values at which the material loses competitive ability compared to commonly used building insulation materials. Specific heat capacity decreased in all cases as a result of low specific heat capacity of salt crystals, which remain in volume of impregnated samples after retardants have dried.

The samples impregnated with ISONEM^®^ ANTI-FIRE SOLUTION and subsequently air-conditioned, contain small crystals that can be seen. The thermal conductivity of crystalline substances is generally high and could have caused a significant increase in the thermal conductivity of the samples. In the case of HR Prof, an oily liquid remains in the samples, which does not dry out even after a long time. The oil coating creates new contacts between the fibres of the material, which leads to an increase in the thermal conductivity of the samples. ECOGARD^®^ B45 impregnation most significantly increases the density of samples and material. No physical changes are visible in the samples with ECOGARD^®^ B45, which prevents the formation of salts, too.

The reason behind the differences of thermal conductivity are the method of retardant application. In the case of spraying, only a thin surface layer of retardant is formed; in the case of dipping, the retardant penetrates the entire sample volume.

The research results of other authors who have looked at thermophysical properties of the thermal insulating materials from recycled waste show that thermal conductivity values of textile waste materials are generally low. The thermal conductivity coefficient for composite panels made of wool waste fibres was determined in range of 0.049–0.060 W·m^−1^·K^−1^ [18]. The material made from residual waste during textile manufacture had thermal conductivity of 0.033–0.039 W·m^−1^·K^−1^. Hadded et al. and Valverde et al. studied the insulation panels made from waste generated during cutting materials in the clothing industry. These panels had a thermal conductivity of 0.041–0.053 W·m^−1^·K^−1^ [46,47]. Gounni et al. found that thermal conductivity of material produced by pulping from recycled acrylic textile waste was 0.038 W·m^−1^·K^−1^ [16]. Material made of almonds shells had thermal conductivity coefficient in the range of 0.074–0.082 W·m^−1^·K^−1^ [8]. A group of materials based on polyurethane foam with an admixture of various materials (recycled rubber crumbs, flax fibres, textile waste) reaches thermal conductivity in the range of 0.027–0.040 W·m^−1^·K^−1^ [40].

Cascone at al. shows that the dry straw bales have an average thermal conductivity of k = 0.0573 W·m^−1^·K^−1^, and their density is around 80 kg·m^−3^. In addition, straw bale walls have good steady thermal performance, but they still lack sufficient thermal inertia, as witnessed by the low phase shift and the high periodic thermal transmittance [48].

## 4. Conclusions

This research focused on the comparison of acoustic and thermophysical properties of Senizol AT XX2 TL60 panels produced from recycled technical textiles before and after application of liquid flame retardants ISONEM^®^ ANTI-FIRE SOLUTION, ECOGARD^®^ B45 and HR Prof, as well as woven and non-woven carbon fibre. The results of sound absorption coefficient measurement showed that these modifications do not have a relevant impact on the researched material. The material was still able to absorb sound in frequency range of 350 Hz to 2 kHz. For easier mutual comparison, sound absorption of the material was also assessed by a single-digit value of NRC, which reached a value of 0.75 after all modifications. Senizol AT XX2 TL60 achieves stable and high sound absorption coefficient after modifications, and according to the standard ISO 11654:1997 it can be classified in sound absorption class A [36].

The research of Senizol AT XX2 TL60 thermophysical properties proves that the material in its original form reaches low thermal conductivity coefficient value at level of 0.038 W·m^−1^·K^−1^, which ranks it among materials with very good insulating properties. Even after application of flame retardants by spraying, the material reaches a low value of thermal conductivity (0.039–0.049 W·m^−1^·K^−1^) and is usable as an insulating material with very good insulating properties, although increase in thermal conductivity occurred at level of 3–30%. After full-volume application of flame retardants by soaking, there is a significant deterioration in thermal conductivity (increase by 2.6–105.3%). The material loses competitiveness against commonly used construction insulation materials after the application of flame retardants ISONEM^®^ ANTI-FIRE SOLUTION and HR Prof. Based on the research results, it is clear that a small change in thermophysical properties was achieved by ECOGARD^®^ B45 retardant, which keeps very good insulating properties of the material in both types of application.

## Figures and Tables

**Table 1:** Thermophysical properties of Senizol AT XX2 TL60 before and after modification with flame retardants.

	*ρ/*(kg∙m^−3^)	*λ/*(W m^−1^∙K^−1^)	*a·*10^7^/(m^2^∙s^−1^)	*c/*(J kg^−1^∙K^−1^)
Ref—without modification	53.4 ± 3.1	0.038 ± 0.001	4.53 ± 0.19	1559.4 ± 72.0
ISONEM^®^ ANTI-FIRE SOLUTION (spraying)	59.8 ± 5.7	0.042 ± 0.001	- *	- *
ECOGARD^®^ B45 (spraying)	58.9 ± 4.1	0.039 ± 0.001	- *	- *
HR Prof (spraying)	58.4 ± 2.1	0.049 ± 0.003	- *	- *
ISONEM^®^ ANTI-FIRE SOLUTION (dipping)	89.3 ± 7.9	0.070 ± 0.002	5.96 ± 0.36	1316.4 ± 44.5
ECOGARD^®^ B45 (dipping)	98.6 ± 9.9	0.047 ± 0.001	2.94 ± 0.34	1311.7 ± 81.7
HR Prof (dipping)	97.7 ± 7.2	0.078 ± 0.002	5.33 ± 0.15	1498.5 ± 91.9

*—EDPS method does not allow to determine values of stated quantities for materials significantly inhomogeneous in direction of sample thickness (inhomogeneity was achieved by applying retardant by spraying on one side of sample surface).

## Data Availability

Not applicable.
